# Diurnal effects of polypharmacy with high drug burden index on physical activities over 23 h differ with age and sex

**DOI:** 10.1038/s41598-022-06039-4

**Published:** 2022-02-09

**Authors:** Trang Tran, John Mach, Gizem Gemikonakli, Harry Wu, Heather Allore, Susan E. Howlett, Christopher B. Little, Sarah N. Hilmer

**Affiliations:** 1 Laboratory of Ageing and Pharmacology, Kolling Institute, Faculty of Medicine and Health, Royal North Shore Hospital, University of Sydney, St Leonards, Sydney, NSW 2065 Australia; 2 Departments of Clinical Pharmacology and Aged Care, Royal North Shore Hospital, St Leonards, Sydney, NSW 2065 Australia; 3 Department of Internal Medicine, Yale University, New Haven, CT 06510 USA; 4 Department of Biostatistics, Yale School of Public Health, New Haven, CT 06510 USA; 5 Department of Pharmacology and Medicine (Geriatric Medicine), Dalhousie University, Halifax, NS B3H 2E1 Canada; 6 Raymond Purves Bone and Joint Research Laboratory, Kolling Institute, Institute of Bone and Joint Research, Royal North Shore Hospital, University of Sydney, St Leonards, Sydney, NSW 2065 Australia

**Keywords:** Preclinical research, Risk factors

## Abstract

Aging, polypharmacy (concurrent use of ≥ 5 medications), and functional impairment are global healthcare challenges. However, knowledge of the age/sex-specific effects of polypharmacy is limited, particularly on daily physical activities. Using continuous monitoring, we demonstrated how polypharmacy with high Drug Burden Index (DBI—cumulative anticholinergic/sedative exposure) affected behaviors over 23 h in male/female, young/old mice. For comparison, we also evaluated how different drug regimens (polypharmacy/monotherapy) influenced activities in young mice. We found that after 4 weeks of treatment, high DBI (HDBI) polypharmacy decreased exploration (reduced mean gait speed and climbing) during the habituation period, but increased it during other periods, particularly in old mice during the transition to inactivity. After HDBI polypharmacy, mean gait speed consistently decreased throughout the experiment. Some behavioral declines after HDBI were more marked in females than males, indicating treatment × sex interactions. Metoprolol and simvastatin monotherapies increased activities in young mice, compared to control/polypharmacy. These findings highlight that in mice, some polypharmacy-associated behavioral changes are greater in old age and females. The observed diurnal behavioral changes are analogous to drug-induced delirium and sundowning seen in older adults. Future mechanistic investigations are needed to further inform considerations of age, sex, and polypharmacy to optimize quality use of medicines.

## Introduction

Physical function is an important global health outcome in old age^1^, with age-related physiological changes and increased vulnerability to multimorbidity contributing to physical/cognitive impairments^2^. Age-associated decline in physical performance often occurs early in the sixth decade of life and can lead to decreased independence and increased frailty^3^. Polypharmacy (concurrent use of ≥ 5 different medications) has emerged as a major healthcare challenge for older people^4^. Polypharmacy is often used to treat multimorbidity but is associated with functional/cognitive impairments^5^, and other adverse outcomes including falls, hospitalization^6^, frailty^7^, and mortality^8^. The effects of polypharmacy are thought to depend on exposure factors (e.g. drug type, dose, duration, combinations)^9^ and demographics (e.g. age, sex, multi-morbidities, frailty)^10^. Investigation of the interactions between sex, gender and polypharmacy has recently been identified as a key knowledge gap in the literature^11^.

It is ethically problematic to conduct interventional studies evaluating polypharmacy in older adults. Therefore, our knowledge of polypharmacy effects has been mainly derived from observational studies. Residual confounding in observational research (e.g., different disease severity, medication indications, heterogeneous onset/duration) impacts the evaluation of the relationship between age, sex, polypharmacy, and function^12^. Observational studies of the association of polypharmacy with physical performance in later life have predominantly evaluated function using questionnaires, short physical tests (e.g. walking speed, grip strength) or different basic/instrumental activities of daily living scales^13^. Recent research suggests that continuous monitoring of daily physical activities over longer periods using wearable sensor technology could detect more complex physical changes than traditional methods^14^. Older adults, in particular, could benefit from this^15^, because there are multiple age-related changes in circadian rhythm, leading to several altered body rhythmic characteristics including activity levels^16^, which could be identified with prolonged/continuous observations^17^. These circadian disruptions could also alter drug responses in aging^18^. However, to date, no clinical study has employed continuous monitoring of physical performance to observe changes associated with polypharmacy, age, and sex.

Animal models are largely used to evaluate the impacts and mechanisms of drugs on physical function without residual confounding^19^. This has been explored across different ages/sexes, predominantly using short traditional out-of-cage tests including open field, rotarod and wire hang^20,21^. Non-invasive automated in-cage assessment tools (e.g., Laboratory Animal Behavior Observation Registration and Analysis System—LABORAS, Metris, Netherlands) have now been validated in laboratory animals to constantly monitor various behaviors^22^. This enables extended testing durations and diurnal variation assessments, in home-cage-like environments, without human interference^22^. This method has not previously been used to assess physical activity in polypharmacy-treated mice.

Recently, mice administered polypharmacy with high Drug Burden Index (DBI—measuring an individual’s cumulative exposure to anticholinergic and sedative medications^23^) showed functional impairment in short physical tests, with varying effects in different ages/sexes^24^. Increasing DBI also increased frailty and functional impairments in aged male mice in conventional out-of-cage behavioral experiments^12^. More information is required on how polypharmacy affects daily physical function in homelike environments, such as LABORAS cages, over long periods, including effects across the diurnal cycle.

Recognizing the clinical gap in literature, in this study, we evaluated changes in physical performance over 23 h, using the LABORAS, following 4 weeks of high DBI (HDBI, DBI score 1.6^12^) polypharmacy compared to controls in male and female, young and old mice. We also determined the effects of different drug regimens (4 weeks of HDBI polypharmacy; low DBI (LDBI) polypharmacy, DBI score 0.5^12^; metoprolol monotherapy, and simvastatin monotherapy) on physical performance over 23 h in young male and female mice. Medications studied belong to drug classes commonly prescribed in older adults^24^, and do not require routine dose-adjustment in old age^25^. They have similar pharmacokinetic/pharmacodynamic properties in mice and humans, and are unlikely to cause toxicity when administered alone to healthy animals^25^. Because of some shared metabolic pathways in the liver, there might be some potential interactions between metoprolol, citalopram, and oxycodone in these regimens, however, they are suggested to be minor^26,27^. Additionally, current knowledge on the interactions beyond drug pairs is limited. Overall, this study investigates treatment (polypharmacy, DBI, and monotherapy), age (young/old), and sex (male/female) effects on physical activities over the day/night cycle in a preclinical model.

## Results

### Verification of tolerance to medications

To confirm all animals tolerated the therapeutic concentrations of medications and the experiment, we measured key animal welfare markers. No significant weight loss (Supplementary Fig. 1), or reduction in food/water intake was observed over 23 h during the LABORAS recording, or during the whole 4 weeks of intervention^24^, which are indicators of tolerance issues.

Interestingly, in LABORAS experiments, some animals did eat more food: mice given HDBI polypharmacy had significantly higher food intake than control (Supplementary Fig. 1c,d). Similarly, young females administered HDBI diet consumed significantly more food than young females given other treatments. No significant differences were detected between groups in water intake over 23 h (Supplementary Fig. 1e,f). Together, these outcomes show the animals tolerated the medication regimens and this LABORAS experiment.

The main findings describing treatment effects on behavior, and the interactions with age and/or sex are summarized in Tables 1 and 2, and/or discussed in detail for each time period below.

**Table 1:** Summary of the comparisons between control and HDBI polypharmacy.

	10 am–11 am				11 am–7 pm				7 pm–7 am				7 am–9 am			
	*Trt*	Trt*A	Trt*S	Trt A*S	*Trt*	Trt*A	Trt*S	Trt*A*S	*Trt*	Trt*A	Trt*S	Trt*A*S	*Trt*	Trt*A	Trt*S	Trt*A*S
Distance					↑								↑	↑ O > Y		
Locomotion					↑				↑	↑ O > Y			↑	↑ O > Y		
Speed	↓		↓ F > M		↓		↓ F > M	↓ YF > YM	↓							
Rearing					↑				↑				↑			
Climbing	↓	↓ Y > O	↓ F > M													
Grooming	↓				↓	↓ O > Y			↓	↓ O > Y						
Immobility	↑												↓	↓ O > Y		

↑, ↓: increase or decrease in HDBI polypharmacy, compared to control.

*Y* young; *O* old, *M* male, *F* female, *Trt* indicating the effects of HDBI polypharmacy treatment, Trt*A indicating treatment × age interactions, Trt*S indicating treatment × sex interactions, Trt*A*S indicating treatment × age × sex interactions.

**Table 2:** Summary of the comparisons among all young mice.

	10 am–11 am		11 am–7 pm		7 pm–7 am		7 am–9 am	
	HDBI	LDBI	HDBI	LDBI	HDBI	LDBI	HDBI	LDBI
**Polypharmacy**								
Distance			↑^C^	↑^C^				
Locomotion			↑^C^	↑^C^				
Speed	↓^C^		↓^C^					
Rearing			↑^C^	↑^C^	↑^C^			
Climbing	↓^C^	↓^C^						
Grooming			↓^C^					
Immobility								

	Metoprolol	Simvastatin	Metoprolol	Simvastatin	Metoprolol	Simvastatin	Metoprolol	Simvastatin
**Monotherapy**								
Distance	↑^HDBI^ ; ↑^LDBI^	↑^HDBI^ ; ↑^LDBI^	↑^C^; ↑^HDBI^	↑^C^; ↑^HDBI^				
Locomotion			↑^C^	↑^C^				
Speed			↑^HDBI^	↑^HDBI^				
Rearing	↑^C^	↑^C^	↑^C^	↑^C^	↑^C^			
Climbing	↑^HDBI^ ; ↑^LDBI^	↑^HDBI^ ; ↑^LDBI^	↑^C^; ↑^HDBI^	↑^C^; ↑^HDBI^; ↑^LDBI^				
Grooming								
Immobility			↓^C^; ↓^HDBI^	↓^C^; ↓^HDBI^				

↑^C^, ↓^C^: increase or decrease in treatment compared to control; ↑^HDBI^, ↓^HDBI^: increase or decrease in treatment compared to HDBI polypharmacy; ↑^LDBI^, ↓^LDBI^: increase or decrease in treatment compared to LDBI polypharmacy.

### The habituation period—[10 am–11 am]

**Figure 1:**
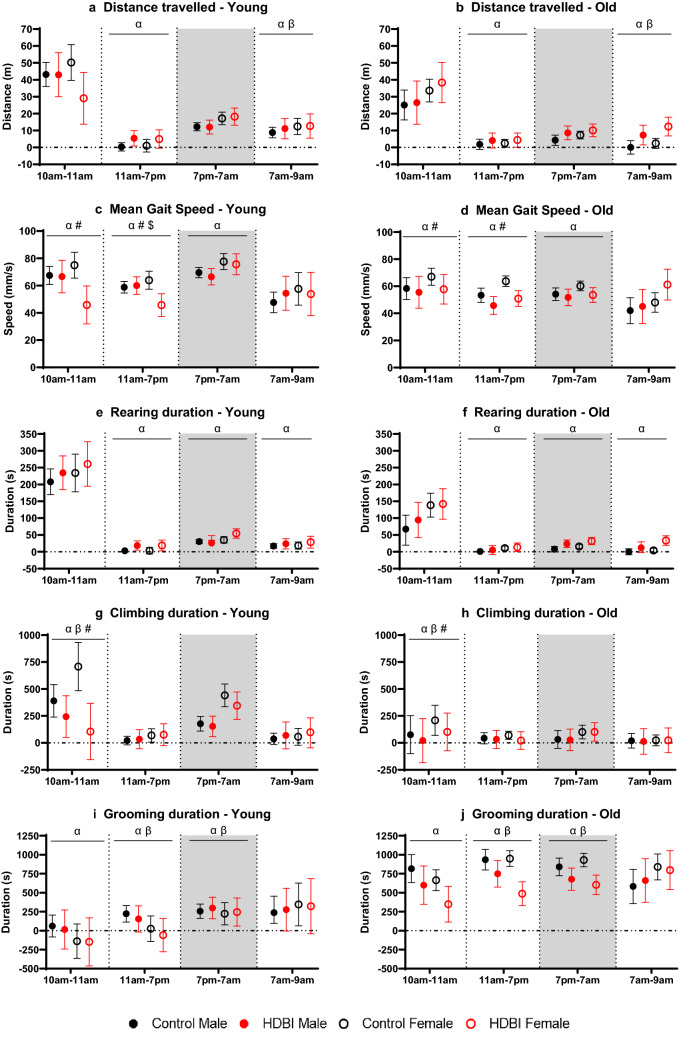
Different physical activities and behaviors in each analyzed period, measured by the LABORAS for control and high DBI polypharmacy regimen in young (5 months old) and old (24 months old) C57BL/6JArc mice of both sexes (*n* = 6–8 per group). (**a,b**) Distance travelled (meters), (**c,d**) mean gait speed (millimeters/second), (**e,f**) duration of rearing (seconds), (**g,h**) duration of climbing (seconds), (**i,j**) duration of grooming (seconds). The results are presented for each outcome and within period as least-squares means and 95% confidence intervals, estimated at the mean body weight. Each period for each activity/behavior was analyzed using a separate linear mixed model with significance based on Type III tests of fixed effects, adjusted for bodyweight and cohort, with Benjamini–Hochberg procedure to adjust for multiple comparisons. The light and shaded area represents the light and dark cycles, respectively. The vertical dotted lines separate different analyzed periods over 23 h. α, p < 0.05, indicating significant treatment effect, comparing all polypharmacy groups to control groups; β, p < 0.05, indicating significant interaction between age and polypharmacy treatment; #, p < 0.05, indicating significant interaction between sex and polypharmacy treatment; $, p < 0.05, indicating significant interaction among age, sex and polypharmacy treatment.

**Figure 2:**
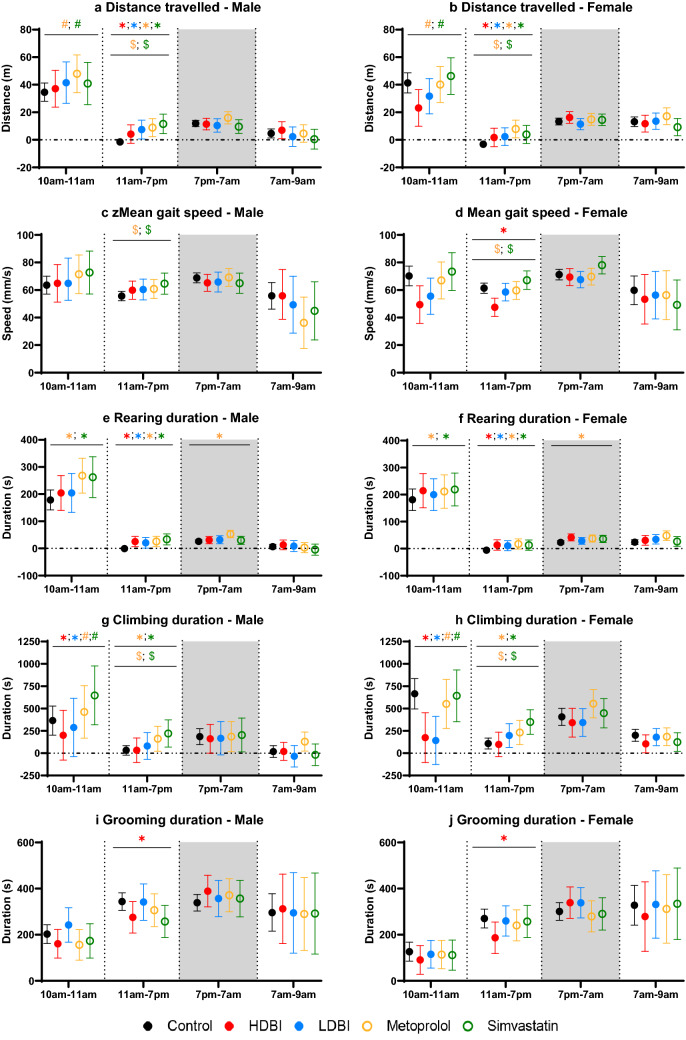
Different physical activities and behaviors in each analyzed period, measured by the LABORAS for control, treatments with polypharmacy diets and monotherapy diets in young (5 months old) C57BL/6JArc mice of both sexes (*n* = 6 per group). (**a,b**) Distance travelled (meters), (**c,d**) mean gait speed (millimeters/second), (**e,f**) duration of rearing (seconds), (**g,h**) duration of climbing (seconds), (**i,j**) duration of grooming (seconds). The results are presented for each outcome and within period as least-squares means and 95% confidence intervals, estimated at the mean body weight. Each period for each activity/behavior was analyzed using a separate linear mixed model, adjusted for bodyweight and cohort, with Benjamini–Hochberg procedure to adjust for multiple comparisons. Significance is based on pairwise comparisons of each treatment to control, or to polypharmacy groups. The light and shaded area represents the light and dark cycles, respectively. The vertical dotted lines separate different analyzed periods over 23 h. *, p < 0.05, for pairwise comparisons between treatment and control, in both sexes; #, p < 0.05, for pairwise comparisons between treatment and both polypharmacy groups, in both sexes; $, p < 0.05, for pairwise comparisons between treatment and HDBI polypharmacy groups, in both sexes (each treatment is represented by a different color in the legend)*.*

To investigate the animals’ ability to cope with a small change in their environments, we considered the first hour of recording to be the acclimatizing period and assessed how the environmental change affected their behaviors. We found that, compared to control, HDBI polypharmacy treatment significantly decreased some spontaneous physical activities in mice of both ages/sexes, including mean gait speed (Fig. 1c,d), climbing (Figs. 1g,h, 2g–h) and grooming time (Fig. 1i,j). Consistent with these decreases in active behaviors, mice given polypharmacy displayed significantly longer immobility time than control during this period (Supplementary Fig. 2c,d).

Significant treatment × sex interactions were detected for mean gait speed and climbing, whereby females declined more after HDBI treatment than males (Fig. 1c,d,g,h). Additionally, when comparing the effect of age and treatment on climbing, there was a greater reduction in climbing time in young compared to old mice in HDBI groups (Fig. 1g,h), indicating a significant age × treatment interaction.

We further analyzed a wider range of treatments in young animals, and found that during the habituation period, mice given LDBI polypharmacy also climbed significantly less than control (Fig. 2g,h). Compared to control, metoprolol and simvastatin monotherapy significantly increased rearing durations in mice of both sexes (Fig. 2e,f). Additionally, compared to both polypharmacy regimens, mice on monotherapies also travelled significantly further (Fig. 2a,b), with longer climbing time (Fig. 2g,h) during the habituation period. No treatment × sex interaction was found among young animals.

In brief, HDBI polypharmacy decreased exploration during the habituation period in all mice, especially in females. Comparisons among young mice also highlighted the reduced climbing time associated with LDBI polypharmacy, compared to control. In contrast, both monotherapies increased exploration in mice, relative to control and polypharmacy.

### The light cycle/Inactive phase—[11 am–7 pm]

We next sought to investigate whether mouse behaviors were altered during the light cycle in the LABORAS cages, following drug treatment. As mice are nocturnal, this represents a period of less activity, equivalent to night-time for humans.

Compared to control, HDBI mice significantly increased some physical behaviors, including distance travelled (Figs. 1a,b, 2a,b), locomotion (Supplementary Figs. 2a,b, 3a,b) and rearing durations (Figs. 1e,f, 2e,f), during 11 am–7 pm. In contrast, mean gait speed (Figs. 1c,d, 2d) and grooming time (Figs. 1i,j, 2i,j) were significantly decreased in mice following HDBI polypharmacy, compared to control.

Similar to the habituation period (10 am–11 am), significant treatment × sex interaction was found for mean gait speed, with a greater decline in HDBI polypharmacy females than in males (Figs. 1c,d, 2d). In young mice, females travelled slower speeds than males after HDBI treatment, indicating a significant treatment × age × sex interaction (Fig. 1c). Additionally, for grooming, a significant age × treatment interaction was found, in which HDBI-associated decrease in grooming time was greater in old mice, compared to young animals (Fig. 1i,j).

Further analyzing in young mice, we found that LDBI polypharmacy, and monotherapy of metoprolol or simvastatin also significantly increased distance travelled (Fig. 2a,b), durations of locomotion (Supplementary Fig. 3a,b) and rearing (Fig. 2e,f), compared to control. Additionally, mice administered metoprolol or simvastatin climbed significantly more than control (Fig. 2g,h), while having significantly longer distance travelled, faster mean gait speed and longer climbing time (Fig. 2a–d,g,h) than HDBI mice. Consistent with the increased exploration described, monotherapy mice displayed shorter immobility durations than control or HDBI groups (Supplementary Fig. 3c,d).

To summarize, during this light period, HDBI polypharmacy increased distance travelled and active time in mice, irrespective of age/sex, while decreasing speed and grooming, with greater declines seen in females and old mice. Among young animals, both monotherapy groups displayed more activity than control or HDBI groups.

### The dark cycle/active phase—[7 pm–7 am]

Next, we explored if treatments affected behaviors during the dark cycle. This period is when mice are commonly awake, equivalent to daytime for humans. Overall, old mice were less active than young mice during this cycle (Fig. 1).

Similar to the effects of HDBI polypharmacy seen in the initial light cycle, during the dark cycle, compared to control, HDBI treatment significantly increased locomotion (Supplementary Fig. 2a,b) and rearing durations (Fig. 1e,f), while decreasing mean gait speed (Fig. 1c,d) and grooming time (Fig. 1i,j).

The increase in locomotion time, after HDBI treatment, was greater in old animals, compared to young mice (Supplementary Fig. 2a,b), indicating a significant treatment × age interaction. In contrast, old mice administered HDBI polypharmacy had a greater reduction in grooming activity than the young (Fig. 1i,j), demonstrating a significant treatment × age interaction.

Next, analyzing among young mice, we found that, compared to control, metoprolol significantly increased rearing time in both sexes (Fig. 2e,f). No treatment × sex interaction was found for any outcomes during this period.

In brief, HDBI polypharmacy increased active time in mice of both sexes, compared to control, with greater increases seen in old mice; and also decreased mean gait speed and grooming. However, despite these treatment-associated increases, overall, old mice were still less active than the young groups.

### The transition to inactive phase—[7 am–9 am]

Finally, we explored the proceeding light cycle, a period when mice will have less activity. In this transition period, we observed that, compared to control, HDBI polypharmacy increased distance travelled, and durations of rearing and locomotion in mice of both ages and sexes (Fig. 1a,b,e,f; Supplementary Fig. 2a,b). This, consequently, also significantly decreased immobility time (Supplementary Fig. 2c,d).

Significant treatment × age interactions were found for distance travelled, locomotion and immobility time. After HDBI treatment, old mice had significantly greater increases in distance travelled and locomotion time, while displaying greater decline in immobility durations than the young (Fig. 1a,b; Supplementary Fig. 2).

Among young animals, no additional treatment effect or treatment × sex interaction was detected during this transition period. Thus, during the transition, HDBI polypharmacy generally increased activity measures in mice regardless of sex, an effect that was less pronounced in younger mice.

## Discussion

Knowledge on age- and sex-specific effects of polypharmacy is limited, particularly on daily physical activities. In the present study, for the first time, we comprehensively explored the impacts of polypharmacy on various spontaneous physical behaviors in mice of different ages/sexes, over prolonged periods including their active/inactive phases. In home-like LABORAS cages, a HDBI polypharmacy regimen decreased exploration during the habituation period, increased some spontaneous physical activities/active durations during the light/dark cycles and during the transition to the next inactive light cycle in male/female mice of both ages. The magnitude of these effects varied between young and old mice. Consistently throughout most of the testing duration, mice given HDBI treatment also displayed slower speeds than the control groups. Interestingly, some polypharmacy-related behavioral decreases (climbing, and mean gait speed) were greater in females than in males. In young animals, compared to control, metoprolol and simvastatin monotherapy increased several physical activities, mainly during the light cycle, however, showed no effect on speed, in contrast to the overall decline observed with HDBI polypharmacy constituting these medications in combination. Taken together, our findings demonstrate that the impacts of polypharmacy on physical activities differ based on age, sex and over a 23-h timeframe.

Polypharmacy-associated declines in physical behaviors were observed during the habituation period of 10 am–11 am, regardless of age/sex. Treated mice exhibited declines in mean gait speed, and the durations of climbing and grooming, compared to control. Climbing is associated with escape attempts, coping mechanisms to captivity-induced stress, curiosity, or a repetitive motor routine to explore^28^. This shows that HDBI polypharmacy inhibited the previously reported increased exploration in control mice during the first hour in LABORAS cages^29^. Relatively novel environments can increase curiosity and/or anxiety in mice^30^. In our study, these behavioral measures may have been abolished by the anxiolytic effects of citalopram in HDBI polypharmacy^31^, leading to reduced activities. This is further supported by the reduced grooming observed in HDBI mice. Grooming is a stress-relieving behavior in animals^32^, and can increase when rodents are in anxiety-provoking situations^33^. Additionally, our mice may also experience drowsiness, dizziness, and reduced balance as adverse effects of long-term exposure to anticholinergic and sedative medications^34,35^, contributing to the reduced mean gait speed and climbing.

These findings align with previous work reporting significant decline in physical activities and exploration following different polypharmacy combinations in young^36,37^ and old mice^12,25^, compared to control using traditional behavioral tests during the light cycle. Particularly, a recent study investigating the same HDBI polypharmacy regimen on the same cohorts of mice as the current study has described similar treatment-related reductions in both sexes in several conventional out-of-cage physical measures, including open field, rotarod, forelimb grip strength^24^. Interestingly, that study reported higher, not lower, anxiety levels in mice administered HDBI polypharmacy, as mice spent much smaller proportions of time in the midzone during open field testing. The discrepancy between our results and earlier work^24^ might be due to some different anxiety-related parameters measured (*grooming time versus midzone time*) and experimental conditions: shorter testing durations, different light settings, environments, and the experimenters were present in the room with the mice in the previous study, contrasting with our work. It is possible that this HDBI polypharmacy can cause heightened anxiety in mice in completely novel environments, leading to lesser exploratory activity as seen during 5-min open field assessment, but, in more familiar surroundings with longer testing time, this regimen displays overall anxiety-relieving effects as detected in our study. However, unlike open field testing^38^, the LABORAS has not been validated to investigate anxiety in rodents, despite its advantages of automated monitoring in homelike conditions. Future research directly comparing these tests is required to confirm the observed findings.

Unlike the initial inhibitory effects, HDBI polypharmacy significantly increased some exploratory behaviors during other analyzed periods, with the consistent decrease in mean gait speed throughout most of the 23-h timeframe, compared to control of both ages/sexes. Increased distance travelled and active durations during the light cycles of 11 am–7 pm (represents the period of resting/less activity for mice) and 7 am–9 am (represents the transition from active to inactive phase for mice) contrast with previous studies assessing polypharmacy effects during the light cycle^12,25,36,37^. Apart from the testing conditions different to earlier work, the observed results could be due to the reduced sleeping behavior in HDBI-treated mice. Medications used in this combination have been previously reported to worsen sleep characteristics when administered chronically^39–42^, which could account for the increased behaviors detected here in mice, when they are typically inactive^43^. Moreover, the increased behaviors during 7 am–9 am were more pronounced in aged HDBI mice, indicating significant treatment × age interactions. This supports the view that old animals might have poorer sleep quality^29,44^ and can sleep less during the beginning of the light cycle than the young^45^, which may have been exacerbated by the HDBI polypharmacy used here.

Additionally, increased eating and drinking during the second half of 7 pm–7 am and during 7 am–9 am (Supplementary Figs. 4 and 5), mainly observed in old animals, might have also contributed to increased activity in mice administered HDBI polypharmacy during the transition period, irrespective of age/sex. This could slightly increase drug levels, thereby potentially influencing behaviors due to delirium from cumulative anticholinergic load^46^, or to the locomotor activating effects of acutely administered oxycodone, leading to increased exploratory behaviors^47^. However, this did not increase mean gait speed or behaviors requiring greater muscle strength or fitness including climbing, potentially because of the dizziness and lack of coordination associated with anticholinergics and sedatives^35^. Interestingly, monotherapy with citalopram in different doses did not increase locomotion and rearing during the transition period in a rat model of 25-h monitoring in an open field^31^. In young animals, differing from the effects of HDBI polypharmacy, LDBI polypharmacy groups did not display any behavioral differences compared to control during 7 am–9 am. This demonstrates the impact of increasing the anticholinergic/sedative burden of a polypharmacy regimen on physical activities.

Increased activities during 7 am–9 am seen in aged mice may also be analogous to the “sundowning” described in some older people with cognitive impairment, who experience agitation, anxiety in the late afternoon and evening as compared to other times of the day^48^. So far, the exact causes of this phenomenon still remain unclear, despite the intensive research into its mechanisms to improve diagnostic and preventive measures for older adults^49^. Sundowning has been investigated mechanistically using transgenic mice, focusing mainly on changes in circadian rhythms of locomotor activity and affective components (e.g. stress, fear and anxiety)^43^. Additionally, anticholinergic medications can precipitate delirium and interfere with the sleep–wake cycle^50^, which might also contribute to the increased behaviors detected here. Our LABORAS experiments have proven to be sensitive to capture the sundowning-related behavioral alterations in old mice following HDBI polypharmacy treatment. Combined with other anxiety assessments, this may be a useful method to comprehensively assess how polypharmacy may lead to or even exacerbate sundowning in old age, and also to investigate preventive/therapeutic options for this syndrome in the setting of polypharmacy and aging.

During the active dark cycle of 7 pm–7 am, there were also some polypharmacy-associated increases in behavioral measures in mice, compared to control of both ages/sexes. HDBI mice displayed longer active time of locomotion and rearing, however, unlike the light cycles, they did not travel significantly further than the control groups. Combined with the observed decreased mean gait speed, it is possible that the increased active duration is more likely the result of mice moving more slowly than control animals, not because they were more active.

This current study also found significant treatment × sex interactions during the habituation period and the light cycle of 11 am–7 pm in the LABORAS, whereby young females had greater declines in mean gait speed than males, following HDBI polypharmacy treatment. Additionally. females but not males climbed significantly less than control during the habituation hour. This is inconsistent with the performance of the same HDBI polypharmacy treated animals in the open field recorded over 5 min and grip strength device, showing no treatment × sex interaction for gait speed, and reduced forelimb grip strength in males and not in females, respectively^24^. This may be due to the presence of different sex effects seen using different tests. One possible explanation for the above differences in reported outcomes, is that the LABORAS apparatus might be more similar to the home-cages, therefore can detect behavioral changes under less stress than the open field. It is also possible that the devices measure different entities of grip strength. The grip strength device measures overall grip strength while the LABORAS assesses climbing behavior.

The mechanisms responsible for the observed sex differences are poorly understood. They could stem from variations in pharmacokinetics/pharmacodynamics between sexes^51^, resulting in females more susceptible to polypharmacy-related adverse events than males^11^. Components of this polypharmacy combination, when administered as monotherapy, have displayed sex-specific pharmacokinetic and pharmacodynamic differences in clinical and preclinical studies. For example, women experience higher drug exposure to metoprolol than men, due to increased absorption, lower volume of distribution, and slower hepatic metabolism via cytochrome P450 2D6 (CYP2D6)^52^. Simvastatin used chronically exerts equally effective cardio-protection in both sexes^52^; however, it can further increase risk of statin-induced myopathy in older females, compared to males^53^. In mouse models of Alzheimer’s disease, citalopram improves spatial learning in female^54^, but not in males^55^. Oxycodone can acutely increase exploratory behaviors in mice of both sexes^47^. When given together, this drug combination could affect the activity of or saturate hepatic cytochrome enzymes such as CYP3A4 and CYP2D6 (shared metabolic pathways of metoprolol, citalopram, and oxycodone), further altering the pharmacokinetics and outcomes^56^.

Metoprolol and simvastatin monotherapy generally increased behaviors in young male/female mice, in contrast to the effects of HDBI polypharmacy that included these drugs, on exploration during the habituation period, and on mean gait speed throughout most of the recording. Mice given LDBI combination displayed more similarities in physical activities to control animals, compared to the HDBI groups. This again highlights that polypharmacy regimens with different anticholinergic/sedative load can have different effects on daily physical function. It is not well understood why the monotherapies used in this study could increase behaviors in young mice, particularly during the light cycle. Beta-blockers have been shown to improve maximal/submaximal exercise capacity in heart failure^57,58^. It is possible that metoprolol also displayed cardioprotective effects in our healthy young mice, therefore increasing physical capacity. However, this is inconsistent with some studies reporting the opposite effects of beta-blockers in healthy mice^59^. Additionally, monotherapy with simvastatin or metoprolol here might have negatively impacted sleeping behaviors in young mice, including difficulties in initiating and maintaining sleep^41,60^, hence the increased physical activities. Further studies are needed to investigate whether the increased behaviors with metoprolol, and simvastatin, compared to HDBI polypharmacy, are maintained in older males and females. Also, comparing anticholinergic and sedative monotherapy effects with their effects in polypharmacy regimens may elucidate synergistic and monotherapy-driven effects.

This is the first animal study to demonstrate how HDBI polypharmacy can affect spontaneous physical activities in mice of varying ages/sexes over 23 h utilizing an automated recording system resembling home-cage environments. It explores male–female differences with polypharmacy and age, an important area of literature where further information is required. Here, we demonstrate how polypharmacy may lead to greater declines in some activities in females than males. Moreover, using non-invasive continuous recording cages throughout both light/dark cycles, we have detected different effects of this HDBI polypharmacy on activities to those detected previously using short traditional tests^24^, which varied between young and old, males and females. These findings also highlighted the importance of considering the time of day in experiments measuring functional outcomes. Our continuous monitoring platforms are comparable to the wearable devices currently being developed/trialed in clinical studies, which may be more beneficial than short physical tests in identifying positive/negative effects of different interventions on physical function over long periods of time^15^. We have also selected clinically relevant polypharmacy regimens and physical measures that can be translated to comparable outcomes in humans. Physical outcomes, including distance travelled, gait speed and rearing, can be related to similar age-dependent locomotor changes in humans^61^. Future studies could increase the recording to several day/light cycles to further investigate the sundowning symptoms observed here and increase habituation time to minimize stress.

This study has some limitations. Firstly, while preclinical models do not have residual confounding often seen in clinical observational studies, healthy animals lack pathologies/diseases commonly encountered in patients. Therefore, we could not evaluate the potentially beneficial effects on function of drug treatment through reducing diseases, or the potentially harmful effects on function through drug-disease interactions in the setting of multimorbidity. Secondly, results observed in our study may be specific to the chosen medications or drug classes only, and not generalizable to other drug classes, doses, or combinations. Thirdly, HDBI feeds appeared to crumble more easily than control feeds, which might have affected the accuracy of food intake measurement after recording. This could lead to HDBI mice being incorrectly evaluated as having consumed more food than other groups. Fourthly, this experiment covered 23 h of the day with no previous acclimatization prior to recording. Therefore, activities during the beginning of the test may be due to the habituation and not representative of this period generally. However, because this affected all animals, we have utilized these hours to assess animals’ ability to handle a small change in their environments. Finally, the single housing condition implemented here to optimize uniform medicated-food access/intake and reduce acute social-isolation stress in LABORAS cages could be a potential confounder for the observed findings. Long-term single-housing of rodents can alter behavioral test outcomes, with some of these being age- and sex-dependent^62^.

To overcome the limitations, future studies could include more drug combinations, increase recording time and design experiments to compare mice administered different polypharmacy regimens, in grouped/socially-housed age- and sex-matched cohorts. It is also crucial to further assess polypharmacy in animal models with diseases to better understand the beneficial or negative consequences of multiple drug uses on different outcomes in the context of multimorbidity, aging and sex. This can contribute to improving translation of animal studies to human research. In addition to physical function, the impacts of polypharmacy, in association with age and sex, should also be preclinically evaluated on cognition, in different organ systems, in healthy conditions and with diseases. Complementary observational studies in humans using wearable sensor technology can determine whether the findings with age, sex, and polypharmacy over the diurnal cycle are also seen in older adults. Interventional randomized studies initiating polypharmacy in young and old people are likely to be limited by ethical and feasibility considerations. However, with enough supporting preclinical and observational data, there may be opportunities to investigate the effects of deprescribing polypharmacy on diurnal activities. Findings from preclinical studies can help inform clinicians of potential adverse events of polypharmacy and their pathogenesis, and guide the optimization of medication use in older adults of both sexes.

In conclusion, this study demonstrates the detrimental effects of HDBI polypharmacy on daily spontaneous physical activities in mice of both ages/sexes during the inactive light cycle, and further extended to the active dark cycle. Some of these impacts tended to be more marked in old age and females, and were comparable to drug-induced delirium and sundowning seen in older adults. Future studies should continue to investigate how polypharmacy can affect different outcomes by age/sex, alongside the mechanisms responsible for these potential interactions.

## Methods

### Study design

This study was designed to discover and characterize the changes in spontaneous physical activities over 23 h in rodents of varying age and sex, following polypharmacy treatment. Our randomized controlled laboratory experiments were conducted in 91 healthy young (4 months) and old (23 months) C57BL/6JArc mice, of both sexes (young males: n = 30; young females: n = 30; old males: n = 16; old females: n = 15). Animals were sourced and housed at the Kearns facility, Kolling Institute, Sydney, Australia. This facility obtains mice from the Animal Research Centre in Perth, WA, Australia and breeds them for up to ten generations to maintain genetic similarity. The sample size was calculated to have enough power to detect a difference in locomotor activity in the open field observed between young and old male mice in our previous study^25^. The numbers in each group were also consistent with previous research testing both sexes in the LABORAS^28^. Behavioral recording experiments were performed before and after four weeks of drug interventions. Only animals that remained alive throughout the whole interventional period and underwent both pre-and post-treatment behavioral assessments were included in the final analysis. All procedures were approved by the Northern Sydney Local Health District’s Animal Care Ethics Committee, Sydney, Australia (RESP/16/348). All experiments were performed in accordance with relevant guidelines and regulations. All authors complied with the ARRIVE 2.0 guidelines.

### Research subjects

Mice of both ages and sexes were randomized from different birth cohorts 2–4 weeks apart. Animals were maintained under controlled environment with a regular 12-h light/dark cycle (lights on 7:00 am; off 7:00 pm) and ad libitum access to food/water. After weaning, mice of the same cohort, age and sex were grouped in cages of up to five, fed standard chow provided by the Kearns facility (Rat and Mouse Premium Breeder Diet; 23% protein; Gordon Specialty Feed, NSW, Australia). At age 2.5 months (young) and 21.5 months (old), mice were individually housed and received non-medicated control feed from Specialty Feeds (Standard Meat Free Mouse and Rat Feed; 20% protein, 4.8% fat, 59.4% carbohydrate, 14 Megajoules/kg; Specialty Feed, WA, Australia). Throughout their lives, mice received environmental enrichment (e.g., a straw, wooden stick, tissue paper) and cages contained a red Perspex nest box.

At age 4 months and 23 months for young and old mice respectively, animals of both sexes were randomized to either continue on non-medicated control feed or change to HDBI polypharmacy feed (same dietary formulation as control feed but with added medications—see Table 3). In additional randomly selected cohorts of young mice of both sexes, LDBI polypharmacy or monotherapy with metoprolol or simvastatin were administered (Table 3). Due to the limited number of old animals, they were only assigned to receive control or HDBI polypharmacy diet. Medication regimens were subsets of those tested in our previous study of chronic administration and deprescribing in aging male mice^12^. The randomization and stratification by age-sex in every cohort were performed using the standard = RAND() function in Microsoft Excel (Microsoft Corp, Washington, USA), as previously described^24^. Each age-sex group included control mice and mice receiving different drug treatments described above (n = 6–8 mice per group). Medications were administered in food and water as in our previous study^12^. Doses were calculated from the minimum effective doses when given as long-term monotherapy to mice^12^.

**Table 3:** Medications including estimated daily dose administered in each polypharmacy and monotherapy group.

Regimen	High DBI polypharmacy	Low DBI polypharmacy	Metoprolol monotherapy	Simvastatin monotherapy
Animals	Young and old mice, of both sexes	Young mice of both sexes	Young mice of both sexes	Young mice of both sexes
Estimated drug dose	Simvastatin (20 mg/kg/day)	Simvastatin (20 mg/kg/day)		Simvastatin (20 mg/kg/day)
	Metoprolol (350 mg/kg/day)	Metoprolol (350 mg/kg/day)	Metoprolol (350 mg/kg/day)	
	Citalopram (15 mg/kg/day)	Citalopram (10 mg/kg/day)		
	Oxycodone (5 mg/kg/day)	Acetaminophen (100 mg/kg/day)		
	Oxybutynin (27.2 mg/kg/day)	Omeprazole (10 mg/kg/day)		

Therapeutic doses were estimated from previous investigations of chronic oral monotherapy of these drugs in mice, based on the observed food intake of 0.11 g food/g body weight/day. Medications in the required doses were mixed with control diet to make up the medicated feeds, or being administered in drinking water (as for oxycodone, to comply with the requirements for safe handling and storing opioid drugs).

*DBI* drug burden index.

### Experimental protocol—behavioral recording using LABORAS platforms

Spontaneous physical activities in all young and old animals were assessed before (baseline assessment) and after 4 weeks of treatment, at age 3 and 22 months (pre-treatment), and at age 5 and 24 months (post-treatment), respectively, using the LABORAS (without acclimatization to the cages). From age 3 to 4 months (young) and 22 to 23 months (old), we performed other pre-treatment behavioral assessments prior to starting treatment (reported previously^24^). All mice remained singly caged from age 2.5 months (young) and 21.5 months (old) until euthanized. The differences between age and/or sex in these cohorts at baseline have been previously described ^29^.

The LABORAS (Release 2.6, Metris, Netherlands) was used to detect and record physical activities automatically, continuously for 23 h, following a protocol previously described^29^. The system operated in a separate single-purpose room in the Kearns facility, with minimal noise and vibration. The room was maintained at the same temperature (19–21 °C) and light/dark cycle as the animals’ daily home cages.

Six animals were tested simultaneously in an individual LABORAS platform/cage each day. The LABORAS recorded 1-h segments over 23 h, from 10 am to 9 am the next day. After each experiment, the cages were cleaned with 70% ethanol before testing the next animal. Physical activities including total distance travelled, mean gait speed, and the durations of locomotion, rearing, climbing, grooming, eating, and drinking were measured. The same paper bedding material as in their home cages was provided (Pura Paper Premium Bedding, Able Scientific, Western Australia, Australia), without nesting material or nest boxes. At least one animal per treatment group was tested per day, with randomization by age and/or sex using the = RAND() function in Microsoft Excel. To minimize external disturbances, no one entered the room once the experiment had commenced.

The recorded behaviors were classified by behavior types as previously described^29^. Behaviors displayed during the first hour of recording (10 am–11 am) were used to evaluate animals’ response to a change in their environment.

### Animal body weight, food intake and water intake

On commencement and after each experiment, body weight, the amount of food and the volume of water were recorded. Changes in body weight, food intake and water intake after 23 h in the LABORAS were calculated by subtracting the post-data from the pre-data.

### Statistical analysis

Four periods were segmented based on the light/dark cycles and active/inactive phases, as observed previously: 10 am–11 am (Habituation period – adjustment to the new environment); 11 am–7 pm (light/inactive); 7 pm–7 am (dark/active); 7 am–9 am (light/transition to inactive)^29^. Each mouse’s hourly individual activities were recorded within the pre-treatment and post-treatment.

Statistical analyses were performed using SPSS Statistics v27 (IBM Corp, New York, USA). For each behavioral outcome for each period, a linear mixed model of within period hourly repeated measures was used, adjusted for the within mouse correlation with a heterogeneous first-order autoregressive covariance structure allowing hourly variation to differ in the pre-treatment and post-treatment observations. Each model was adjusted for bodyweight and birth cohort. The a priori comparisons examined whether the effects of treatment relative to control differed by age and sex combinations with Type III tests of fixed effects and the least-squares means within each period estimated at the mean body weight. All main effects were included, as well as age and sex interactions with HDBI treatment relative to control (i.e., treatment × age, treatment × sex, and treatment × age × sex). In separate similar models for each outcome within each period, of only young mice, the treatment effect of HDBI, LDBI, metoprolol and simvastatin, relative to control, and treatment × sex interactions were estimated.

To control for multiple comparisons across outcomes, significance levels were corrected using the Benjamini–Hochberg procedure with the false discovery rate of 0.1. Results are presented for each outcome and within period as covariate-adjusted least-squares means and 95% confidence interval. Raw hourly data is presented descriptively in the Supplementary section.

## Supplementary Information

Supplementary Figures.

## Data Availability

All data are available in the main text or the supplementary materials. Raw data are available upon request.
