# Supplementary material for: Diurnal effects of polypharmacy with high drug burden index on physical activities over 23 h differ with age and sex
Source: Sci Rep. 2022 Feb 9;12:2168. doi: 10.1038/s41598-022-06039-4 (PMC8828819; doi:10.1038/s41598-022-06039-4)
Supplement: Supplementary file 1 — Supplementary Figures. [file 41598_2022_6039_MOESM1_ESM.pdf]

Diurnal effects of polypharmacy with high Drug Burden Index on physical activities over 23 hours differ with age and sex

Trang Tran[1,2]**, MD, John Mach*[1,2]*, PhD, Gizem Gemikonakli*[1,2]*, BSc (Adv) (Hons), Harry Wu*[1,2]*, MBBS, Heather Allore*[3,4]*, PhD, Susan E. Howlett*[5]*, PhD, Christopher B. Little*[6]*, PhD, Sarah N. Hilmer*[1,2]*, MD, PhD*

[1]Laboratory of Ageing and Pharmacology, Kolling Institute, Faculty of Medicine and Health, University of Sydney and Royal North Shore Hospital; St Leonards, Sydney, New South Wales, 2065, Australia.

[2]Departments of Clinical Pharmacology and Aged Care, Royal North Shore Hospital; St Leonards, Sydney, New South Wales, 2065, Australia.

[3]Department of Internal Medicine, Yale University; New Haven, Connecticut, 06510, United States.

[4]Department of Biostatistics, Yale School of Public Health; New Haven, Connecticut, 06510, United States.

[5]Department of Pharmacology and Medicine (Geriatric Medicine), Dalhousie University; Halifax, Nova Scotia, B3H 2E1, Canada.

[6]Raymond Purves Bone and Joint Research Laboratory, Kolling Institute, Institute of Bone and Joint Research, Royal North Shore Hospital and University of Sydney; St Leonards, Sydney, New South Wales, 2065, Australia.

*Corresponding author: Trang Tran. Email: qtra0373@uni.sydney.edu.au

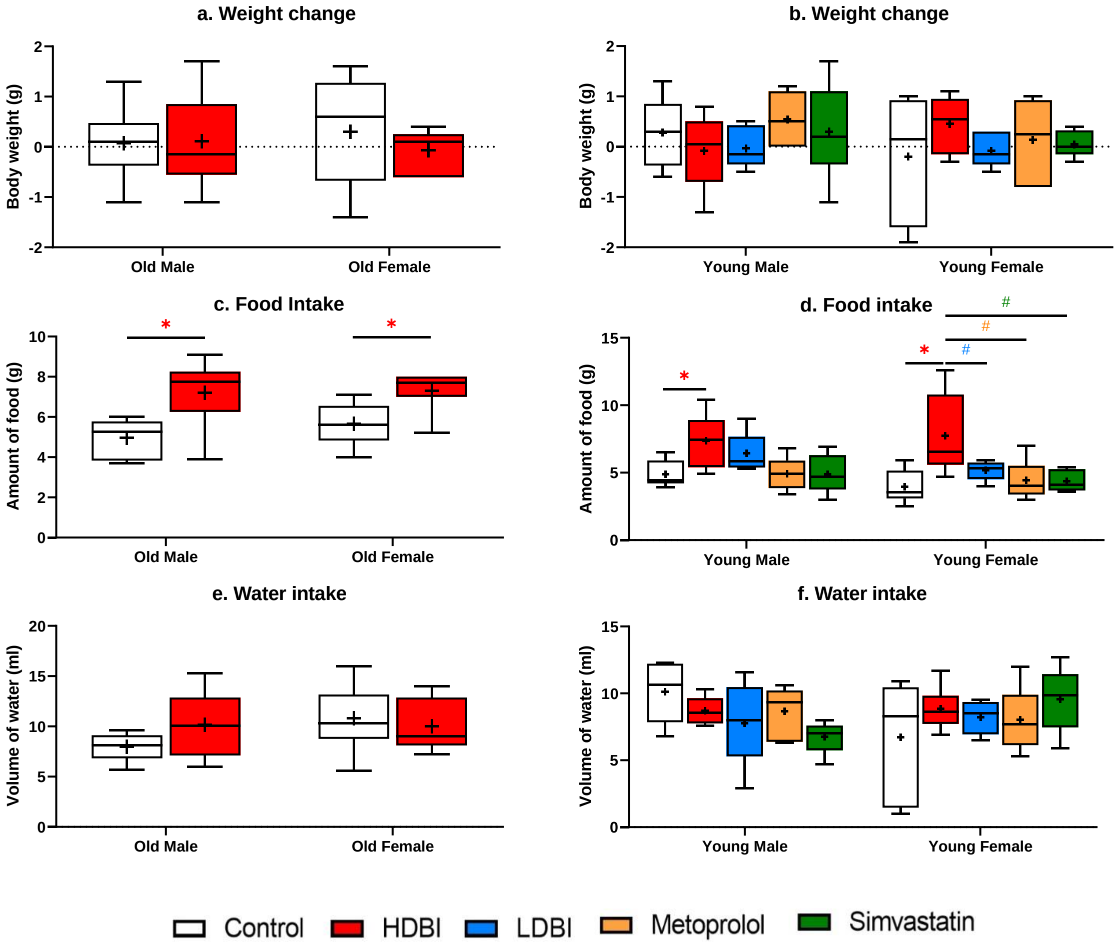

Supplementary Figure 1. Body weight change, food intake, and water intake after 23 hours of LABORAS experiment for control, polypharmacy treatments and monotherapy treatments in young (5 months old) and old (24 months old) C57BL/6JArc mice of both sexes (n = 6-8 per group). (**a**) Weight change in old; (**b**) Weight change in young; (**c**) Food intake in old; (**d**) Food intake in young; (**e**) Water intake in old; (**f**) Water intake in young. Results are presented in a box and whiskers plot. The box contains the 25th to 75th percentiles of the data set; the black line denotes the 50th percentile; "+" represents the mean value; the whiskers mark the 5th and 95th percentiles. Statistical comparisons based on ANOVA.

*, $p<0.05$: statistically significant difference comparing high DBI polypharmacy to control;

#, $p<0.05$: statistically significant difference comparing each treatment to high DBI polypharmacy regimen.

(Each treatment is represented by a different color in the legend).

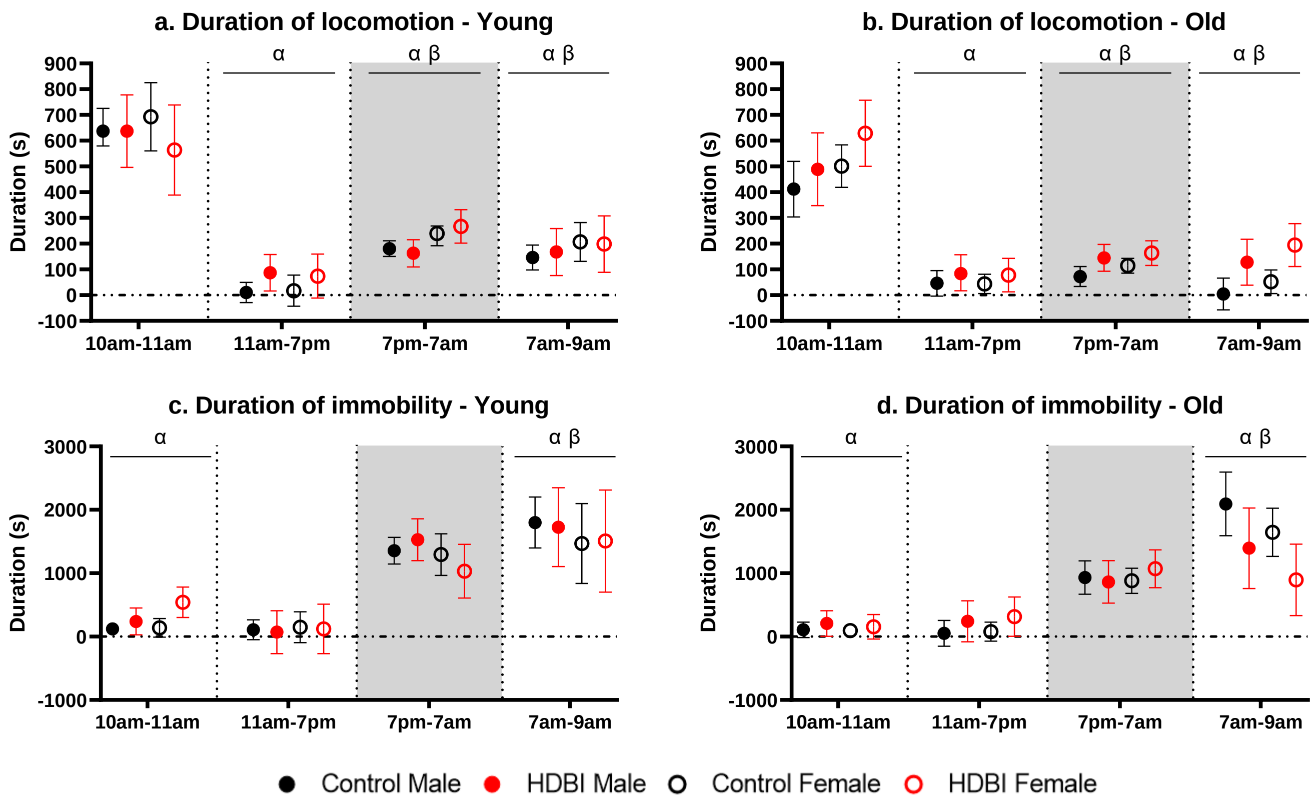

Supplementary Figure 2. Different physical activities and behaviors in each analyzed period, measured by the LABORAS for control and high DBI polypharmacy regimen in young (5 months old) and old (24 months old) C57BL/6JArc mice of both sexes (*n* = 6-8 per group). (**a-b**) Duration of locomotion (seconds), (**c-d**) Duration of immobility (seconds). The results are presented for each outcome and within period as least-squares means and 95% confidence intervals, estimated at the mean body weight. Each period for each activity/behavior was analyzed using a separate linear mixed model with significance based on Type III tests of fixed effects, adjusted for bodyweight and cohort, with Benjamini-Hochberg procedure to adjust for multiple comparisons. The light and shaded area represents the light and dark cycles, respectively. The vertical dotted lines separate different analyzed periods over 23 hours.

α, $p<0.05$, indicating significant treatment effect, comparing all polypharmacy groups to control groups;

β, $p<0.05$, indicating significant interaction between age and polypharmacy treatment.

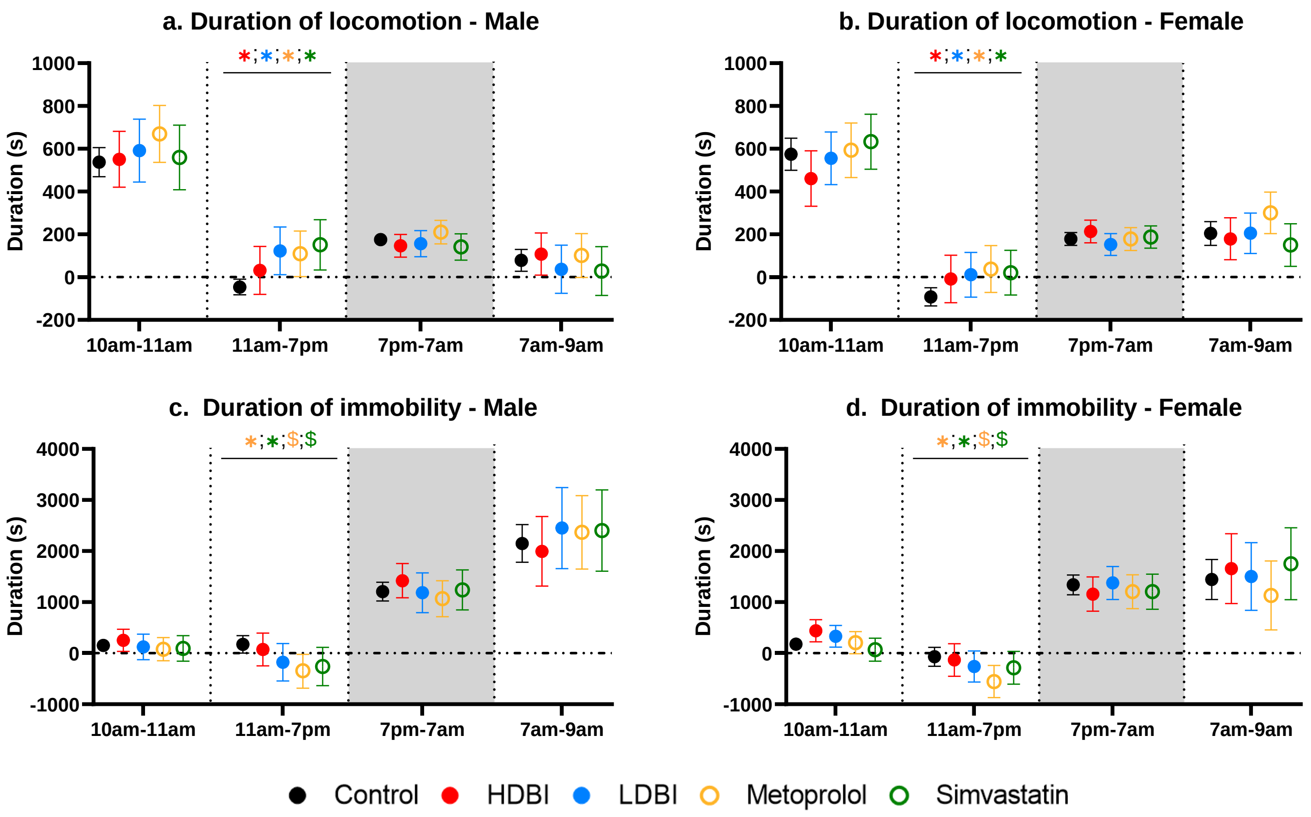

Supplementary Figure 3. Different physical activities and behaviors in each analyzed period, measured by the LABORAS for control, treatments with polypharmacy diets and monotherapy diets in young (5 months old) C57BL/6JArc mice of both sexes (*n* = 6 per group). (**a-b**) Duration of locomotion (seconds), (**c-d**) Duration of immobility (seconds). The results are presented for each outcome and within period as least-squares means and 95% confidence intervals, estimated at the mean body weight. Each period for each activity/behavior was analyzed using a separate linear mixed model, adjusted for bodyweight and cohort, with Benjamini-Hochberg procedure to adjust for multiple comparisons. Significance is based on pairwise comparisons of each treatment to control, or to polypharmacy groups. The light and shaded area represents the light and dark cycles, respectively. The vertical dotted lines separate different analyzed periods over 23 hours.

*, $p<0.05$, for pairwise comparisons between treatment and control, in both sexes;

$, $p<0.05$, for pairwise comparisons between treatment and HDBI polypharmacy groups, in both sexes.

(Each treatment is represented by a different color in the legend).

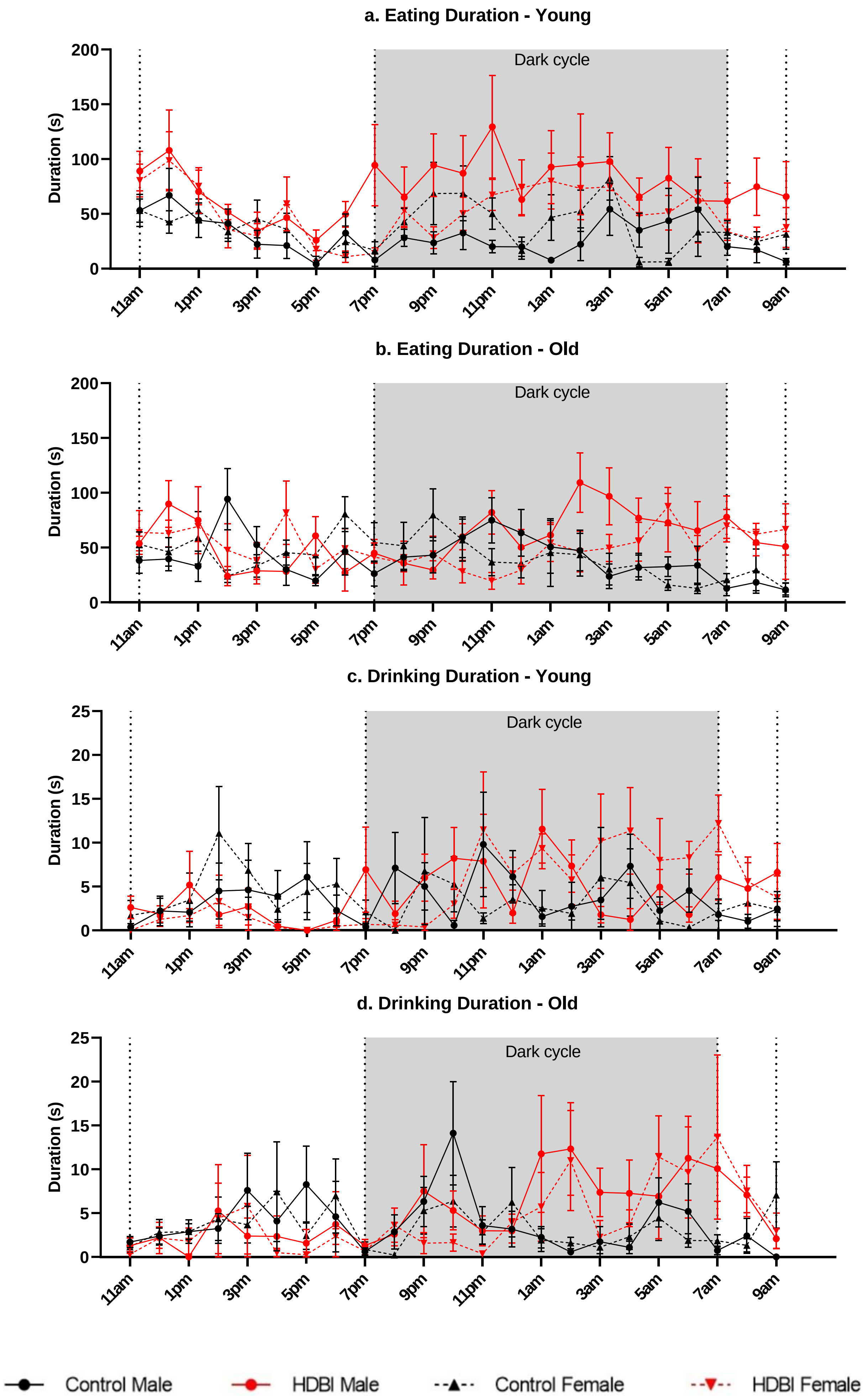
a. Eating Duration - Young
Dark cycle
Duration (s)
200
150
100
50
0
11am
1pm
3pm
5pm
7pm
9pm
11pm
1am
3am
5am
7am
9am
b. Eating Duration - Old
Dark cycle
Duration (s)
c. Drinking Duration - Young
Dark cycle
Duration (s)
25
20
15
10
5
0
d. Drinking Duration - Old
Dark cycle
Duration (s)
Control Male
HDBI Male
Control Female
HDBI Female

Supplementary Figure 4. Durations of eating and drinking in each hour, measured by the LABORAS for control and high DBI polypharmacy regimen in young (5 months old) and old (24 months old) C57BL/6JArc mice of both sexes (n = 6-8 per group). (**a-b**) Duration of eating (seconds); (**c-d**) Duration of drinking (seconds). The results are presented for each hour as observed mean ± SEM for each group. The data point plotted for each hour on the *x* axis represents the outcome recorded from the hour before it to that time point. The light and shaded area represents the light and dark cycles, respectively. The vertical dotted lines represent different periods over 23 hours.

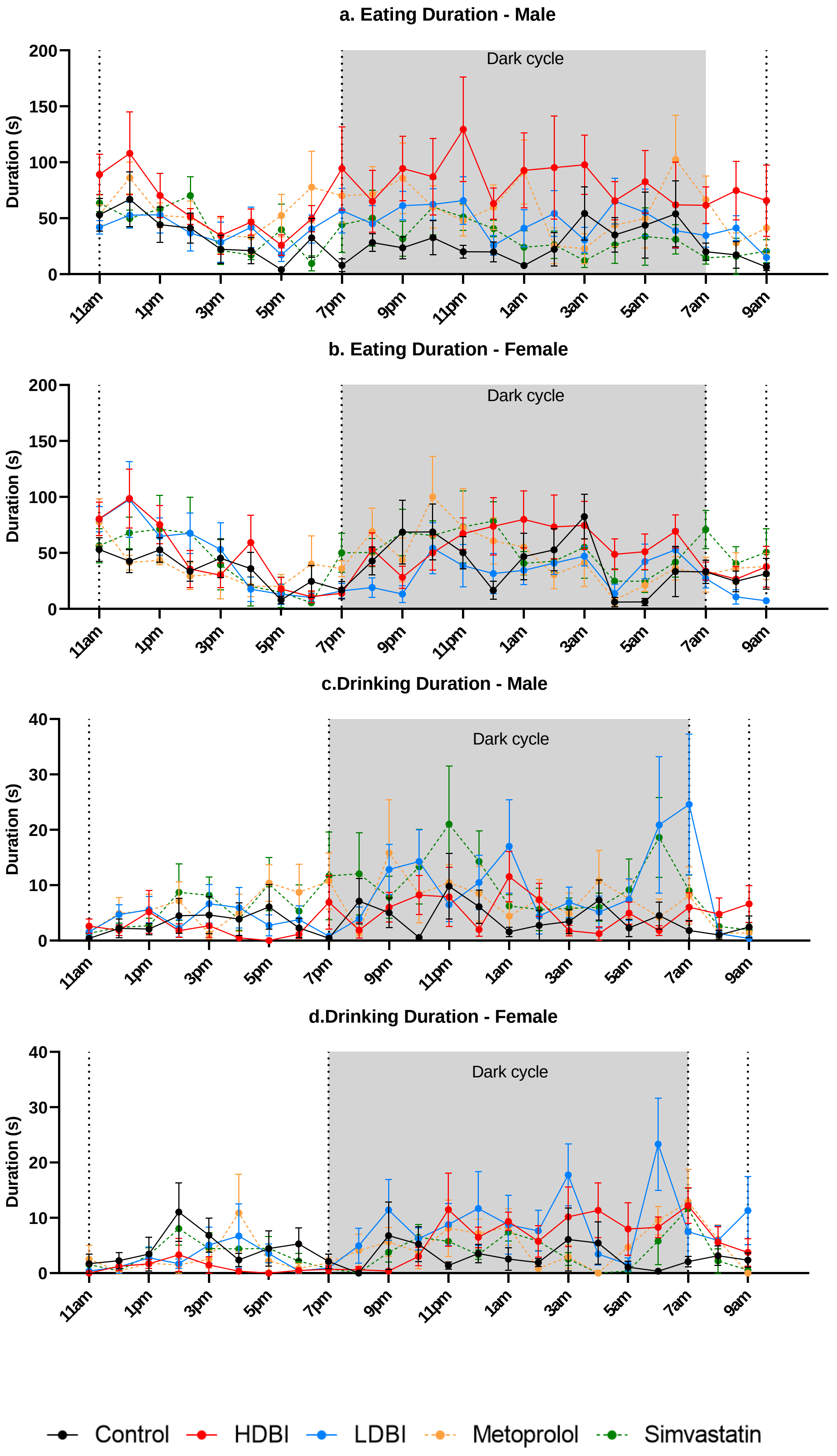
a. Eating Duration - Male
Dark cycle
Duration (s)
200
150
100
50
0
11am
1pm
3pm
5pm
7pm
9pm
11pm
1am
3am
5am
7am
9am
b. Eating Duration - Female
Dark cycle
Duration (s)
200
150
100
50
0
11am
1pm
3pm
5pm
7pm
9pm
11pm
1am
3am
5am
7am
9am
c.Drinking Duration - Male
Dark cycle
Duration (s)
40
30
20
10
0
11am
1pm
3pm
5pm
7pm
9pm
11pm
1am
3am
5am
7am
9am
d.Drinking Duration - Female
Dark cycle
Duration (s)
40
30
20
10
0
11am
1pm
3pm
5pm
7pm
9pm
11pm
1am
3am
5am
7am
9am
Control
HDBI
LDBI
Metoprolol
Simvastatin

Supplementary Figure 5. Durations of eating and drinking in each hour, measured by the LABORAS for control, treatments with polypharmacy diets and monotherapy diets in young (5 months old) C57BL/6JArc mice of both sexes (*n* = 6 per group). (**a-b**) Duration of eating (seconds); (**c-d**) Duration of drinking (seconds). The results are presented as observed mean ± SEM for each group. The data point plotted for each hour on the *x* axis represents the outcome recorded from the hour before it to that time point. The light and shaded area represents the light and dark cycles, respectively. The vertical dotted lines represent different periods over 23 hours.

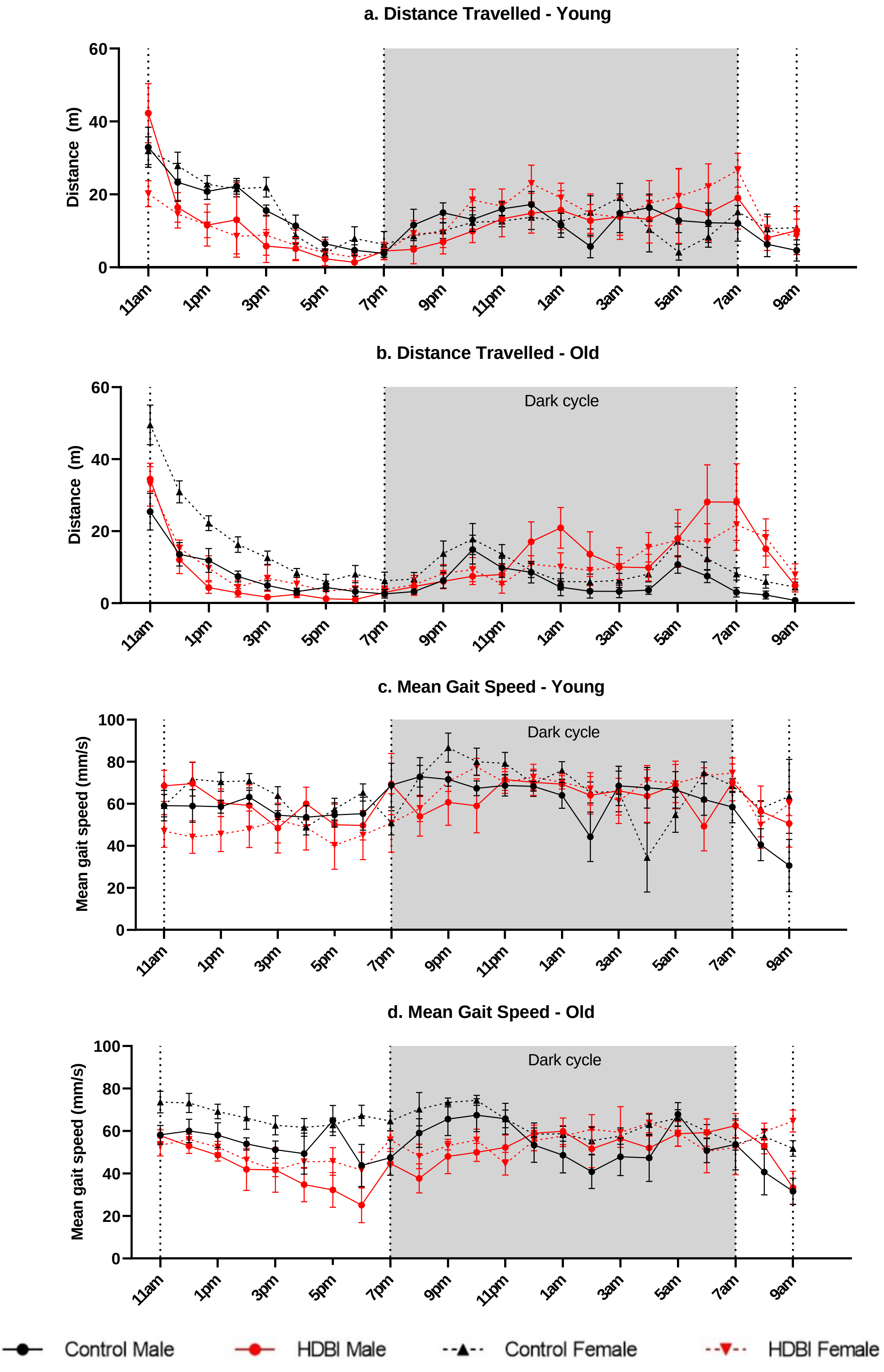
a. Distance Travelled - Young
Distance (m)
b. Distance Travelled - Old
Dark cycle
Distance (m)
c. Mean Gait Speed - Young
Dark cycle
Mean gait speed (mm/s)
d. Mean Gait Speed - Old
Dark cycle
Mean gait speed (mm/s)
11am
1pm
3pm
5pm
7pm
9pm
11pm
1am
3am
5am
7am
9am
Control Male
HDBI Male
Control Female
HDBI Female

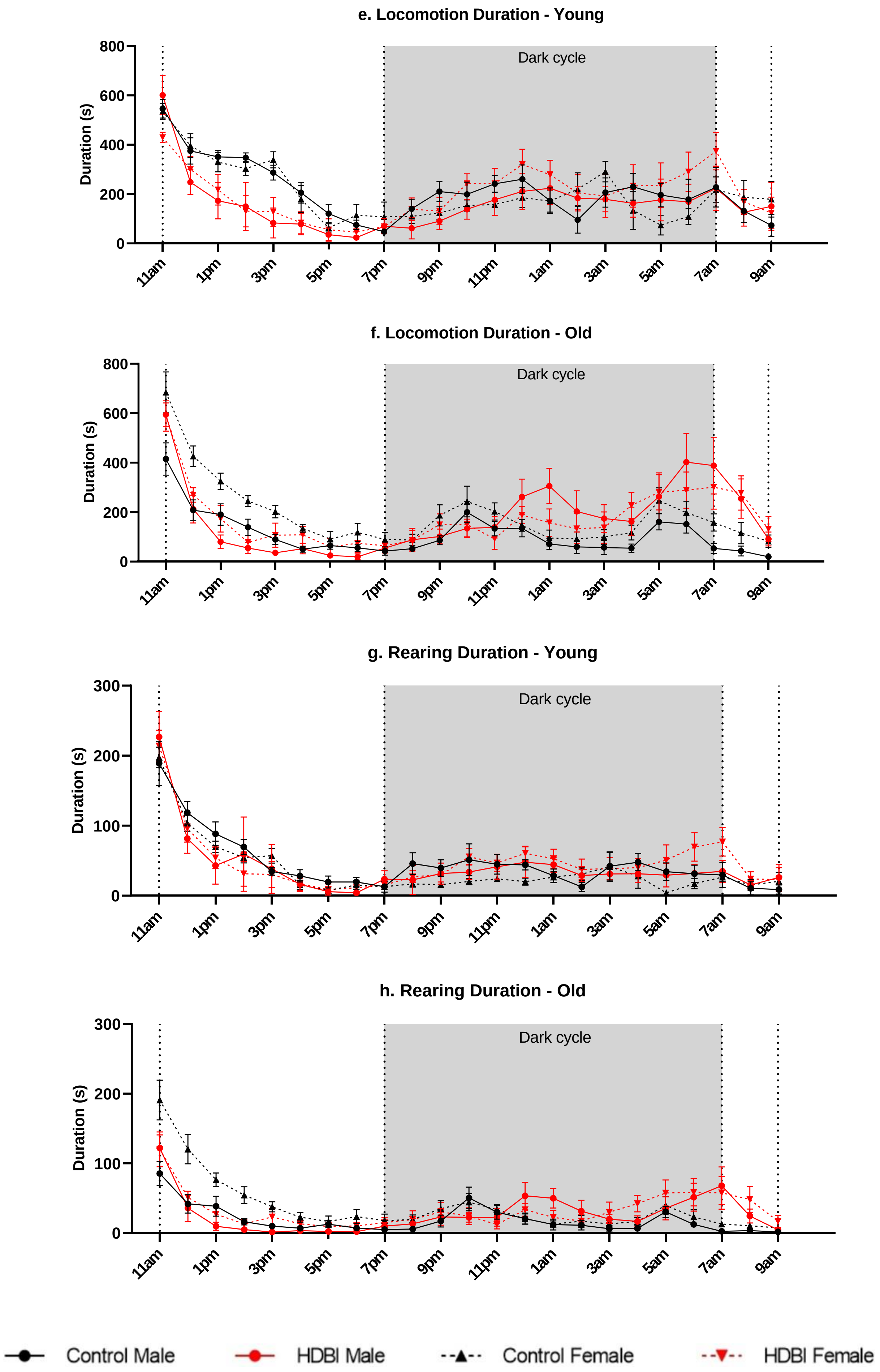
e. Locomotion Duration - Young
f. Locomotion Duration - Old
g. Rearing Duration - Young
h. Rearing Duration - Old
Duration (s)
Dark cycle
11am
1pm
3pm
5pm
7pm
9pm
11pm
1am
3am
5am
7am
9am
Control Male
HDBI Male
Control Female
HDBI Female

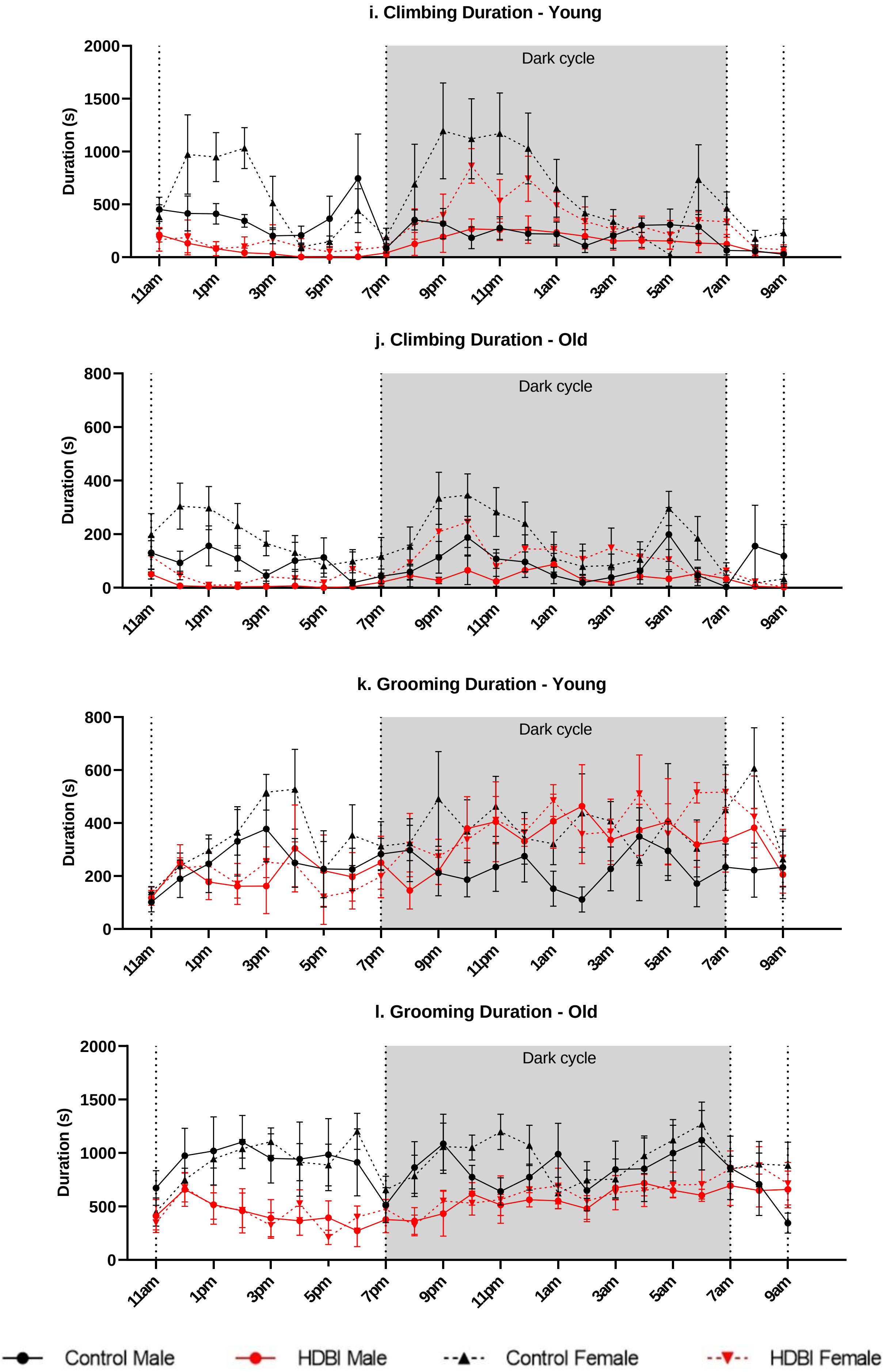
i. Climbing Duration - Young
Duration (s)
2000
1500
1000
500
0
Dark cycle
11am
1pm
3pm
5pm
7pm
9pm
11pm
1am
3am
5am
7am
9am
j. Climbing Duration - Old
Duration (s)
800
600
400
200
0
Dark cycle
11am
1pm
3pm
5pm
7pm
9pm
11pm
1am
3am
5am
7am
9am
k. Grooming Duration - Young
Duration (s)
800
600
400
200
0
Dark cycle
11am
1pm
3pm
5pm
7pm
9pm
11pm
1am
3am
5am
7am
9am
l. Grooming Duration - Old
Duration (s)
2000
1500
1000
500
0
Dark cycle
11am
1pm
3pm
5pm
7pm
9pm
11pm
1am
3am
5am
7am
9am
Control Male
HDBI Male
Control Female
HDBI Female

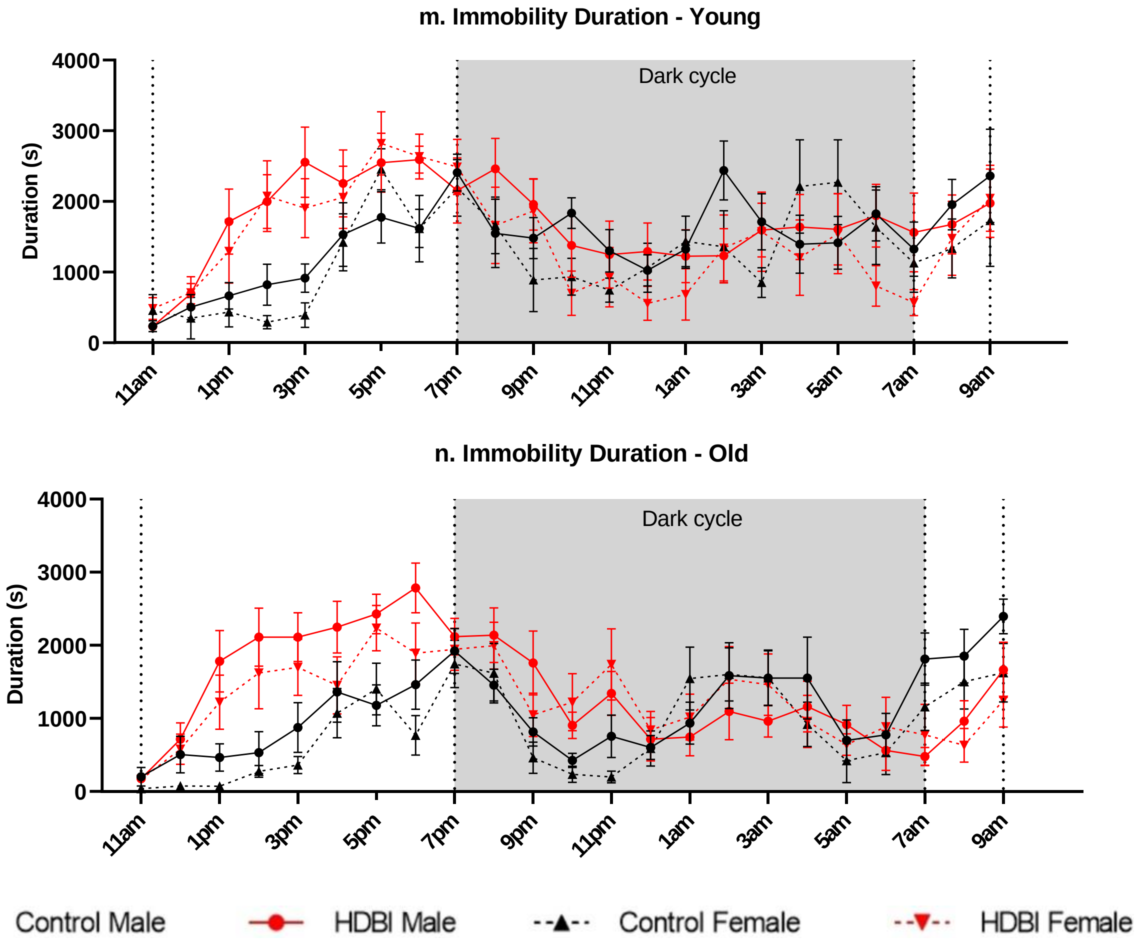

Supplementary Figure 6. Different physical activities and behaviors in each hour, measured by the LABORAS for control and high DBI polypharmacy regimen in young (5 months old) and old (24 months old) C57BL/6JArc mice of both sexes (*n* = 6-8 per group). (**a-b**) Distance travelled (meters), (**b-c**) Mean gait speed (millimeters/second), (**e-f**) Durations of locomotion (seconds), (**g-h**) Duration of rearing (seconds), (**i-j**) Duration of climbing (seconds), (**k-l**) Duration of grooming (seconds), (**m-n**) Duration of immobility (seconds). The results are presented as observed mean ± SEM for each group. The light and shaded area represents the light and dark cycles, respectively. The vertical dotted lines represent different analyzed periods over 23 hours. The vertical axes on (**i-l**) panels are set differently for young and old mice, to account for the differences in climbing and grooming between two age groups.

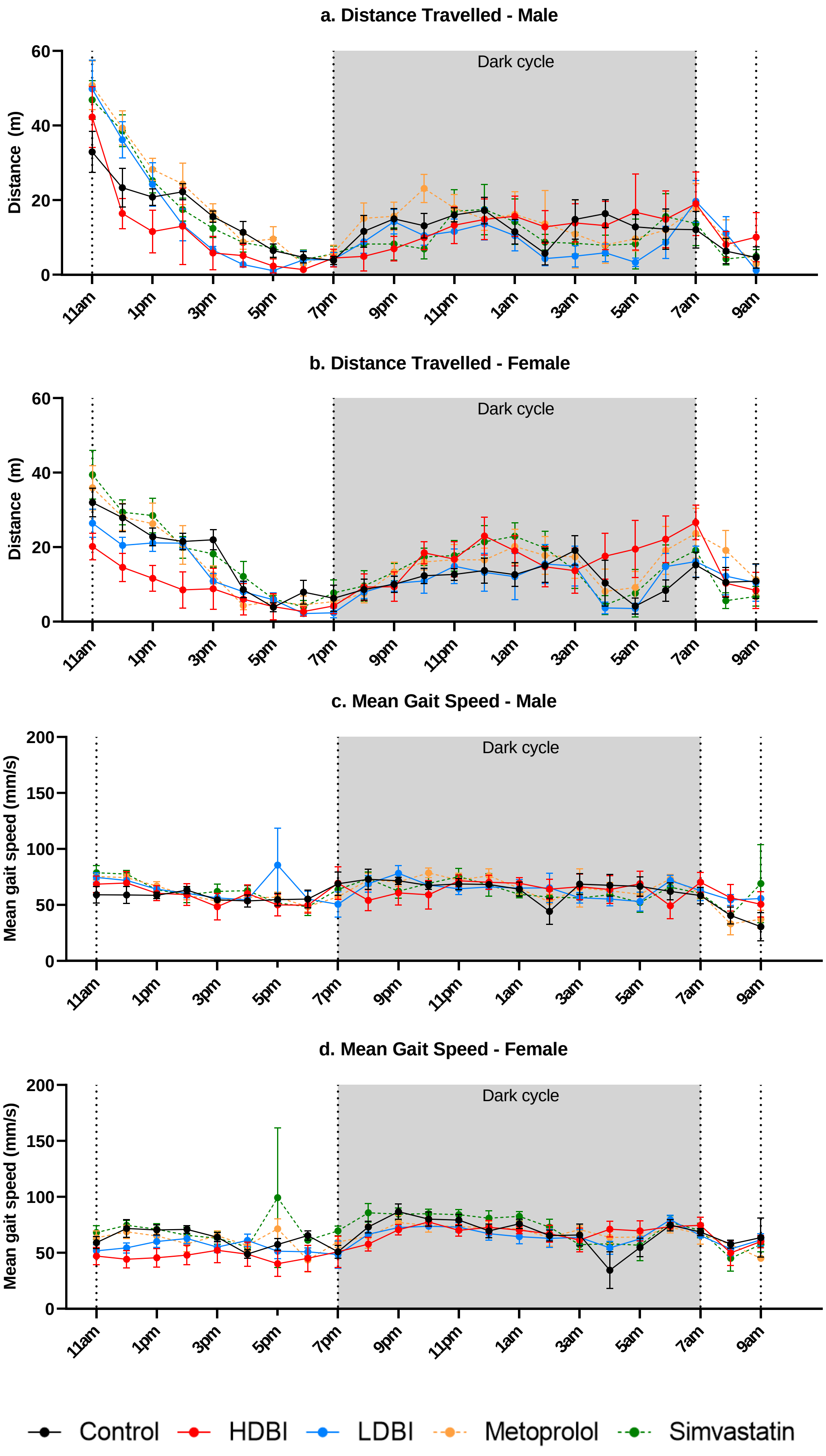
a. Distance Travelled - Male
Distance (m)
Dark cycle
0
20
40
60
11am
1pm
3pm
5pm
7pm
9pm
11pm
1am
3am
5am
7am
9am
b. Distance Travelled - Female
Distance (m)
Dark cycle
c. Mean Gait Speed - Male
Mean gait speed (mm/s)
Dark cycle
0
50
100
150
200
d. Mean Gait Speed - Female
Mean gait speed (mm/s)
Dark cycle
Control
HDBI
LDBI
Metoprolol
Simvastatin

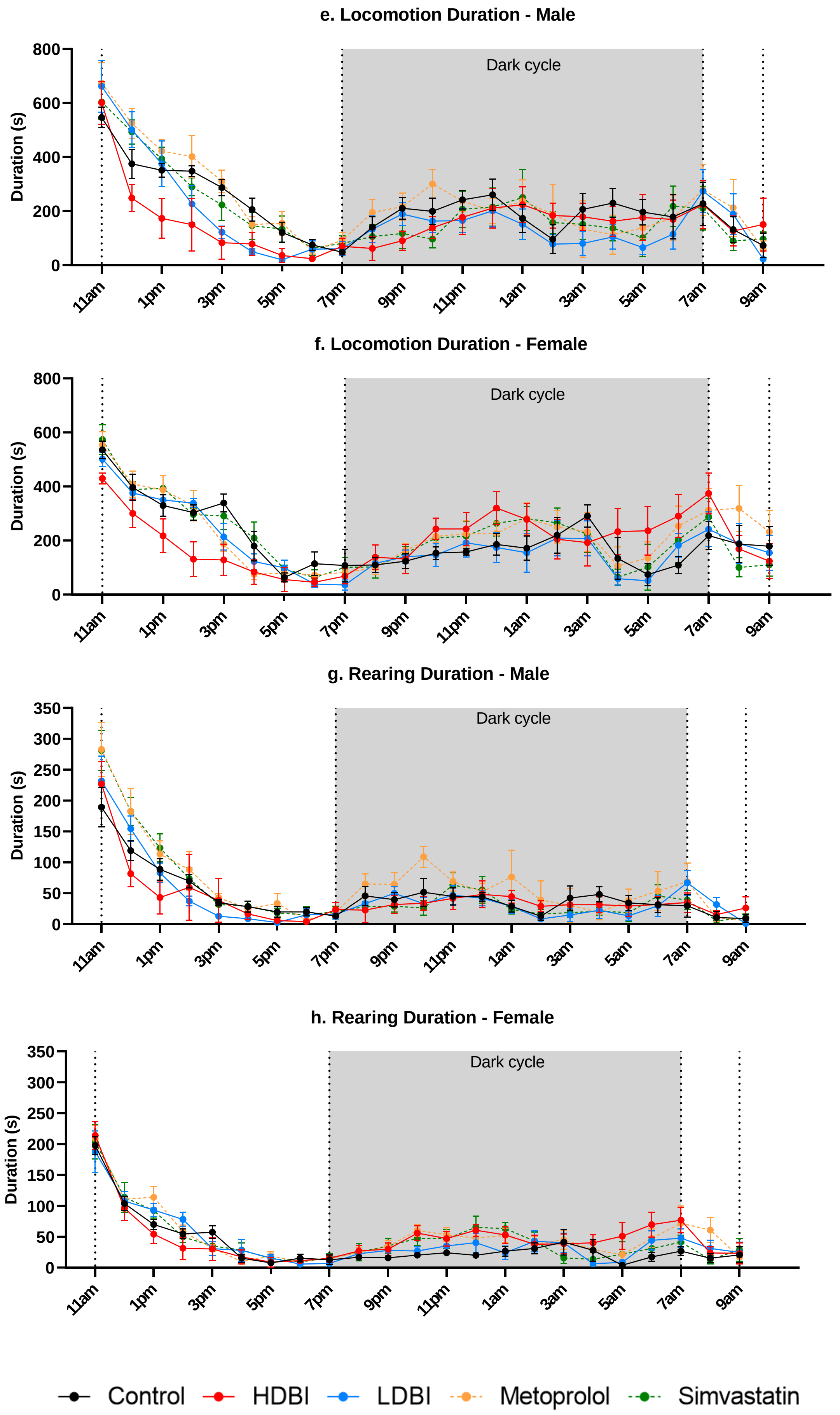

e. Locomotion Duration - Male
Duration (s)
0
200
400
600
800
Dark cycle
11am
1pm
3pm
5pm
7pm
9pm
11pm
1am
3am
5am
7am
9am
f. Locomotion Duration - Female
Duration (s)
0
200
400
600
800
Dark cycle
11am
1pm
3pm
5pm
7pm
9pm
11pm
1am
3am
5am
7am
9am
g. Rearing Duration - Male
Duration (s)
0
50
100
150
200
250
300
350
Dark cycle
11am
1pm
3pm
5pm
7pm
9pm
11pm
1am
3am
5am
7am
9am
h. Rearing Duration - Female
Duration (s)
0
50
100
150
200
250
300
350
Dark cycle
11am
1pm
3pm
5pm
7pm
9pm
11pm
1am
3am
5am
7am
9am
Control
HDBI
LDBI
Metoprolol
Simvastatin

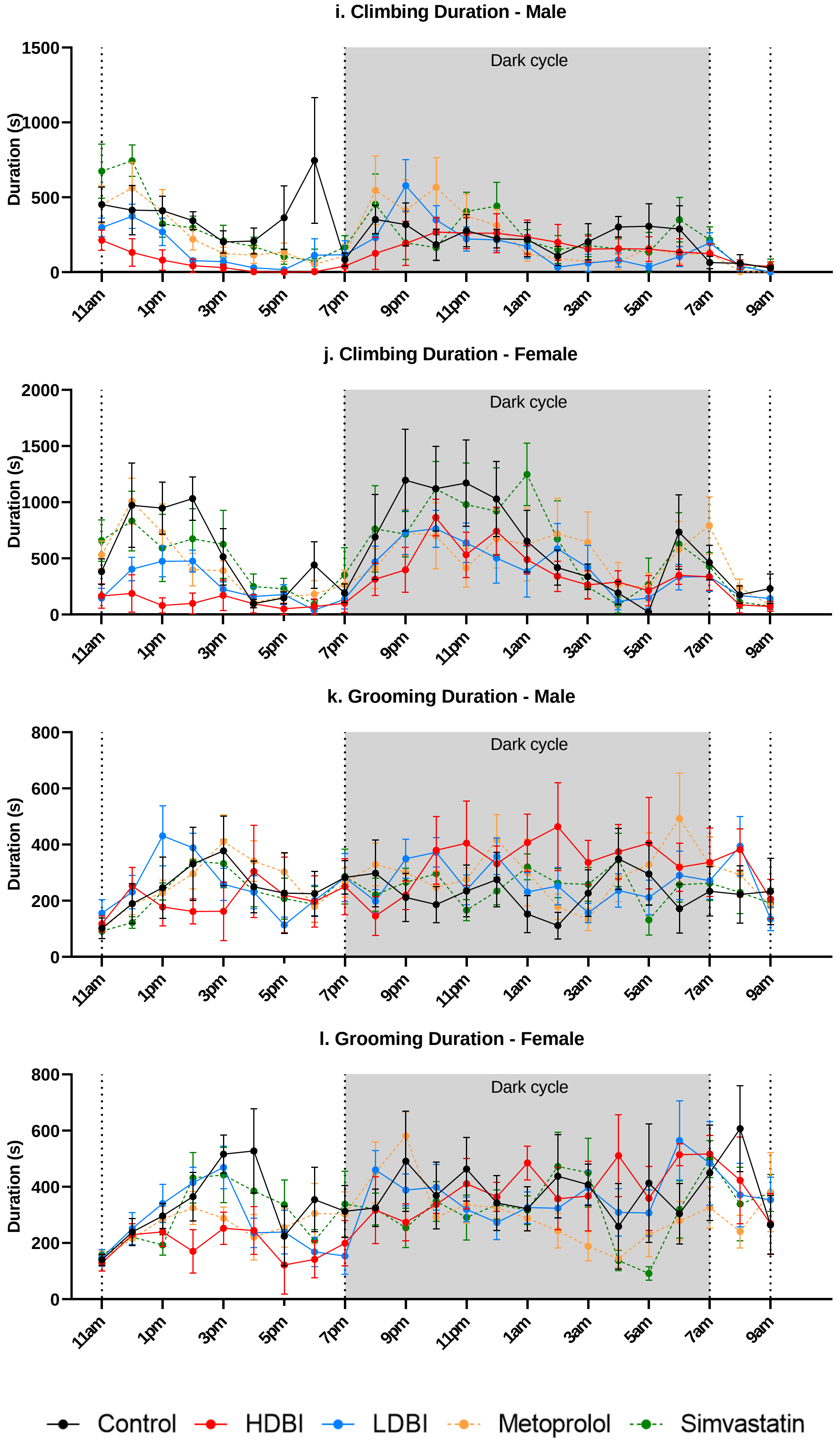

i. Climbing Duration - Male
Dark cycle
Duration (s)
1500
1000
500
0
11am
1pm
3pm
5pm
7pm
9pm
11pm
1am
3am
5am
7am
9am
j. Climbing Duration - Female
Dark cycle
Duration (s)
2000
1500
1000
500
0
k. Grooming Duration - Male
Dark cycle
Duration (s)
800
600
400
200
0
l. Grooming Duration - Female
Dark cycle
Duration (s)
800
600
400
200
0
Control
HDBI
LDBI
Metoprolol
Simvastatin

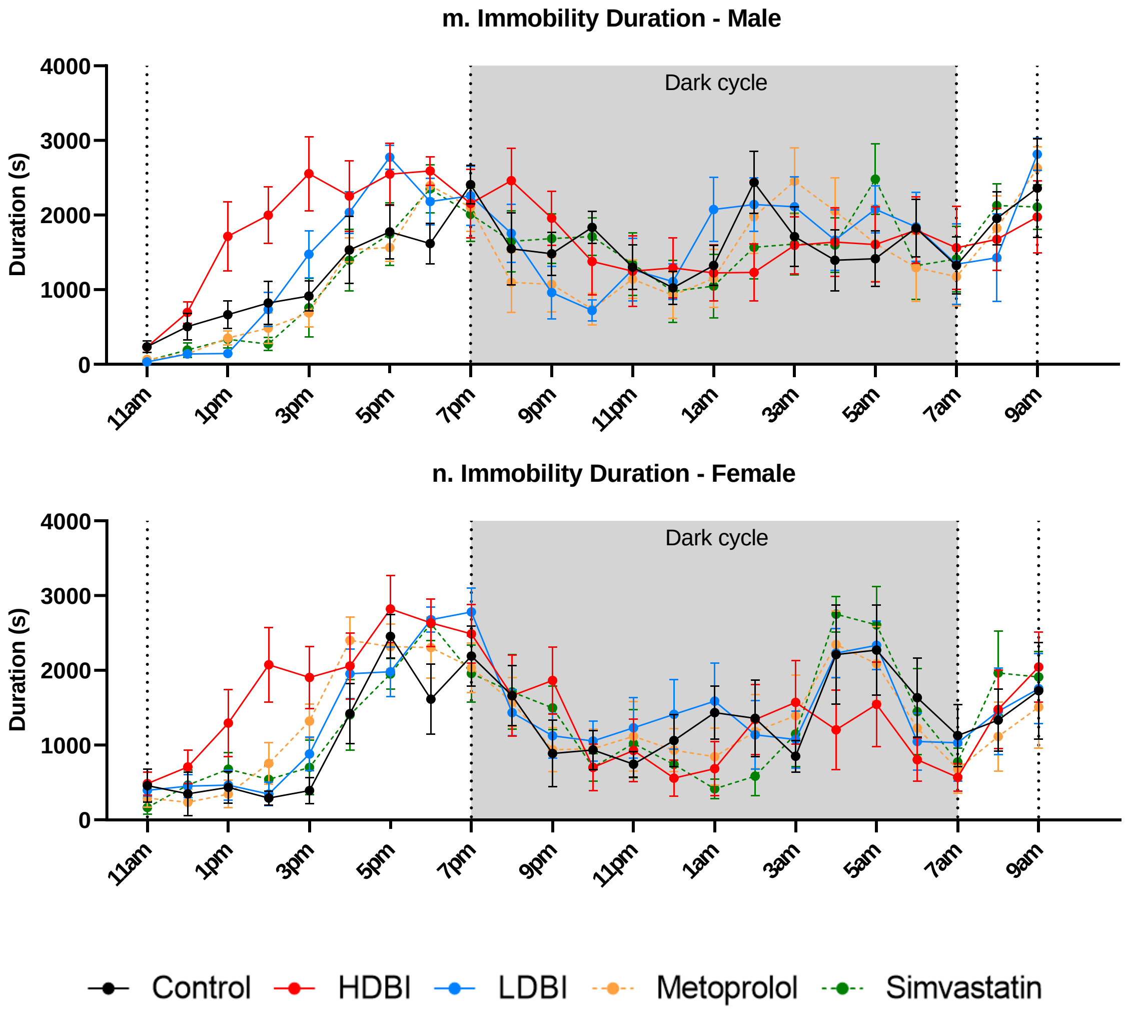

Supplementary Figure 7. Different physical activities and behaviors in each hour, measured by the LABORAS for control, treatments with polypharmacy diets and monotherapy diets in young (5 months old) C57BL/6JArc mice of both sexes (*n* = 6 per group). (**a-b**) Distance travelled (meters), (**b-c**) Mean gait speed (millimeters/second), (**e-f**) Durations of locomotion (seconds), (**g-h**) Duration of rearing (seconds), (**i-j**) Duration of climbing (seconds), (**k-l**) Duration of grooming (seconds), (**m-n**) Duration of immobility (seconds). The results are presented as observed mean ± SEM for each group. The light and shaded area represents the light and dark cycles, respectively. The vertical dotted lines represent different analyzed periods over 23 hours. The vertical axes on (**i-j**) panels are set differently for male and female mice, to account for the differences in climbing and grooming between two sex groups.
